# Biodegradable Active Packaging With Improved Functionality Through PVA, Itaconic Acid, Chitosan, Nanoparticles, and Lemon Peel for Sustainable Solutions

**DOI:** 10.1002/fsn3.70911

**Published:** 2025-09-26

**Authors:** Bernadette‐Emőke Teleky, Silvia Amalia Nemes, Diana Plamada, Laura Mitrea, Calina Ciont, Mădălina Medeleanu, Mădălina‐P. Plosca, Bianca Obreja, Răzvan Odocheanu, Loredana Leopold, Lucian Barbu‐Tudoran, Floricuța Ranga, Dan C. Vodnar

**Affiliations:** ^1^ Institute of Life Sciences University of Agricultural Sciences and Veterinary Medicine Cluj‐Napoca Romania; ^2^ Faculty of Food Science and Technology University of Agricultural Sciences and Veterinary Medicine Cluj‐Napoca Cluj‐Napoca Romania; ^3^ Electron Microscopy Center “C. Craciun”, Biology and Geology Faculty Babes‐Bolyai University Cluj‐Napoca Cluj‐Napoca Romania; ^4^ National Institute for R&D of Isotopic and Molecular Technologies (INCDTIM) Cluj‐Napoca Cluj‐Napoca Romania

**Keywords:** bioactivity, biodegradability, food quality, packaging, shelf life

## Abstract

The growing demand for sustainable and eco‐friendly packaging has driven research into developing biodegradable materials with enhanced functionality. This study develops and evaluates biodegradable polyvinyl alcohol (PVA)‐based films incorporating itaconic acid (IA), chitosan (Ch), lemon peel extract (Lp), and silver nanoparticles (Np's) for active food packaging applications. In a controlled 11‐day storage study at 4°C, when used to coat blueberries, these bioactive films showed reduced weight loss by up to 13.3%, maintained visual quality, and exhibited significantly lower microbial loads (***p < 0.001) compared to uncoated controls. The films exhibited pH‐responsive swelling, peaking at pH 9 (92.95% for PVA + Ch) and reduced water vapor permeability (1.6% ± 1.2% for PVA + IA + Np + Lp). Biodegradability tests revealed up to 61.8% degradation in 8 weeks, confirming their environmental benefits. Adding IA and Ch enhanced mechanical strength and moisture resistance, while Lp and Np's provided antioxidant properties, making these films promising alternatives to conventional plastic packaging. This study presents a scalable approach to developing multifunctional, biodegradable films with potential for real‐world adoption in sustainable food packaging systems.

## Graphical abstract

This study explores biodegradable PVA‐based films enhanced with itaconic acid, chitosan, lemon peel extract, and silver nanoparticles for active food packaging. When applied to blueberries, these bioactive coatings reduced weight loss, maintained visual quality, and limited microbial growth, outperforming uncoated samples. The films also demonstrated pH‐responsive swelling, improved moisture resistance, and 61.8% biodegradation in eight weeks, offering a sustainable alternative to conventional plastic packaging.

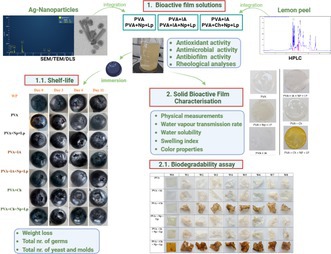

## 1 Introduction

The escalating global concern over plastic pollution and environmental degradation has catalyzed significant research into sustainable food packaging alternatives (do Nascimento et al. 2024; Firdaus et al. 2024). Biopolymers derived from renewable resources present a viable solution due to their biodegradability and lower environmental impact than petroleum‐based plastics. Moreover, the growing recognition of the health implications of packaging materials has driven the shift toward safer alternatives. Petroleum‐based plastics release potentially harmful substances, such as microplastics and chemical additives, into food, posing risks to human health (Hahladakis et al. 2018). Biopolymers, being inherently safer and often biocompatible, address these concerns while aligning with sustainability goals. The COVID‐19 pandemic has further emphasized the necessity for secure and sustainable food packaging, driving advancements in active and intelligent packaging systems (Jayakumar et al. 2023; Pascuta and Vodnar 2022). These systems extend shelf life, maintain food quality, and incorporate sensors for real‐time monitoring, thereby meeting consumer demands and promoting sustainability. The health benefits of these packaging systems are particularly notable, as they protect food from microbial contamination and minimize chemical leaching, ensuring that packaged food remains safe for consumption (Dodero et al. 2021).

Polyvinyl alcohol (PVA) is a widely used biopolymer in packaging due to its non‐toxic, water‐soluble nature and ease of processing (Teleky et al. 2022). These properties have facilitated its application in various fields, particularly in the packaging industry. Additionally, PVA‐based films are recognized for their remarkable mechanical properties and outstanding flexibility, making them ideal for packaging applications, especially in the food industry. Furthermore, the biocompatibility of PVA has enabled its use in producing various biomaterials, expanding its utility beyond traditional packaging solutions. PVA enhances consumer safety, making it a preferred choice in food‐contact materials and reinforcing its role as a safer alternative to conventional plastics (Channa et al. 2022). Building on the strengths of PVA, incorporating itaconic acid (IA) or chitosan (Ch) further enhances the material's functionality by introducing pH‐responsive or antimicrobial properties essential for active packaging applications (Xue et al. 2024). Incorporating (IA) introduces carboxylic acid groups, enhancing the film's pH responsiveness. These groups enable dynamic swelling behavior, crucial for active packaging applications. By accepting protons at low pH and releasing them at high pH, the IA‐modified PVA bioactive films respond dynamically to environmental conditions. This pH‐dependent proton exchange, coupled with the introduction of silver nanoparticles (Np's), positions these bioactive films as promising materials for active packaging, offering antimicrobial properties and improved functionality in various packaging environments (Teleky et al. 2022). Lemon peel (Lp) extract supplies polyphenols and flavonoids that contribute to both antioxidant activity and color modulation. These synergistic interactions enhance the film's overall barrier, mechanical, and bioactive performance.

Integrating Np's into biopolymer films has emerged as a promising strategy to enhance their performance in food packaging applications. Np's significantly improve biopolymer films' barrier and mechanical properties by acting as fillers, making them more resistant to moisture and mechanical stress (Ragab et al. 2024). Additionally, active components in the films provide lasting antioxidant effects that help extend food shelf life. These enhancements reduce spoilage and protect consumer health by limiting microbial proliferation and preserving the nutritional quality of food (Mitrea et al. 2024). Assessing the biodegradability of packaging materials is crucial for understanding their environmental impact, as biodegradable films decompose into non‐toxic byproducts like water and carbon dioxide, avoiding long‐term accumulation in landfills or oceans (Wang et al. 2022). This assessment also ensures that packaging aligns with sustainable development goals by minimizing ecological footprints and promoting a circular economy.

This study is the first to combine IA, Ch, Np's, and Lp extract into a single PVA‐based biodegradable film, creating a multifunctional material that has not been previously reported. The novel inclusion of Lp extract, a sustainable and underutilized bioactive source, further strengthens the film's functionality. Together, these components provide a synergistic solution for active food packaging, offering improved preservation performance and environmental benefits. Blueberries, due to their high moisture content and vulnerability to microbial contamination, require effective solutions to maintain freshness and extend shelf life (Xu et al. 2025). Unlike previous research on plant extracts in PVA/Ch blends (Fakraoui et al. 2024), this study introduces Lp as a natural, eco‐friendly additive known for its bioactive properties. The aim of this research is to develop biodegradable bioactive films with enhanced barrier properties specifically designed to act against moisture and microbial intrusion. Rigorous testing demonstrated the film's effectiveness in preserving blueberries and reducing food waste over 8 weeks, assessed through weight loss and biodegradability in soil. Active components like Np and Lp enhance preservation, supporting sustainable development by promoting environmental conservation and food security. The films' structural, physical, mechanical, and functional characteristics confirm their suitability for various applications in the food industry.

## 2 Materials and Methods

### 2.1 Synthesis and Characterization of Nanoparticles

#### 2.1.1 Synthesis of Nanoparticles

Citrate‐reduced silver colloids (AgNp) were synthesized using the Lee‐Meisel method (Dong et al. 2021). In this process, 90 mL of water with 0.017 g of AgNO_3_ was heated to 100°C until homogenization. Then, 10 mL of an aqueous solution containing 0.020 g of sodium citrate was added gradually with vigorous stirring, and the reaction was allowed to proceed for 24 min. The colloid's pH was adjusted and maintained above 7 using 0.1 M NaOH to ensure colloidal stability during synthesis and characterization.

#### 2.1.2 Dynamic Light Scattering (DLS) and Zeta Potential Measurement

The AgNp were characterized according to their size, polydispersity index (PDI), and Zeta potential using Dynamic Light Scattering (DLS) with a Zetasizer Nano ZS (Malvern Instruments, Worcestershire, UK). The instrument features a 4 mW He–Ne laser operating at a wavelength of 633 nm and an avalanche photodiode detector. Zeta potential measurements were performed using laser Doppler Electrophoresis. All measurements were conducted in triplicate at 25°C. The optical parameters for the analysis were set based on Mie theory, with a refractive index of 1.35 and an absorption index of 3.99 (Leopold et al. 2022).

#### 2.1.3 Morphological Measurements

Transmission electron microscopy (TEM) analysis was performed using a HITACHI HD‐2700 STEM microscope (Hitachi, Tokyo, Japan), integrated with a digital image recording system and an SU8230 scanning electron microscope (SEM) from the same manufacturer. The electron microscope was paired with an Aztec X‐Max 1160 EDX detector (Oxford Instruments, Abingdon, UK) for energy‐dispersive X‐ray spectroscopy (EDS). SEM/EDS images were obtained under 30 kV and 10 μA settings. Samples were mounted on carbon‐coated 400 mesh copper grids, and image acquisition and particle size measurement were conducted using Hitachi acquisition software (version 8.1). The average particle size was calculated using Gaussian fitting in Origin 2019b (Racuciu et al. 2025).

### 2.2 Preparation of Lemon Peel Extract

Untreated lemons, variety Verna, were purchased from a local market (origin La Murada, Alicante). The lemons underwent peeling, drying (40°C for 72 h), and grinding (JustBuy High‐Speed Multifunctional Grinder CE, 3000 W, and a rotation speed of 36,000 rpm) before being prepared for hydrolysis in a 1:5 solution of citrus peel in 50 mM sodium acetate buffer at pH 4.8, following the method of Wilkins et al. (2007) (Wilkins et al. 2007). A pectinase solution was added to facilitate pectin breakdown to achieve a concentration of 1165 IU/g dry matter. The mixture was then sonicated (Elma Schmidbauer GmbH, Singen, Germany) at 20 kHz and 30 W for 30 min, using a 3 mm diameter tip with pulse cycles of 10 s on and 5 s off, while kept in an ice bath to preserve thermolabile phenolic compounds. Afterward, the mixture was centrifuged at 10,000 rpm for 10 min. The supernatant containing the extracted polyphenols was filtered through a 0.45 μm nylon filter, and a 20 μL aliquot was subsequently injected into the HPLC system for analysis. Extraction efficiency was determined based on total phenolic content (7396.77 ± 5.40 μg/mL), quantified using HPLC analysis. Following the method outlined by Nemes et al. (2024), the analysis was conducted using an Agilent Technologies HP‐1200 liquid chromatography system. This setup included a quaternary pump, autosampler, diode array detector (DAD), and an MS‐6110 single quadrupole API‐electrospray detector, ensuring comprehensive detection and quantification of compounds (Nemes et al. 2024).

### 2.3 Preparation of PVA‐Based Films

The bioactive film solutions were prepared by developing a base solution with polyvinyl alcohol (PVA) and glycerol as the control sample. This solution was made with 3% (*w/v*) PVA and Gly (30% w/w relative to PVA dry mass) dissolved in hot distilled water (90°C) under constant stirring for 2 h. Furthermore, the solvent casting technique was used to prepare the polymer films, as indicated in our previous study (Teleky et al. 2022). To the base solution (PVA + Gly) 1% (*w/v*) IA or 1% (*w/v*) Chitosan (Ch) was added under stirring conditions and at a temperature of 65°C for 30 min to obtain the formulation PVA + IA or PVA + Ch. This solution was enriched with 9% lemon peel (Lp) extract and 1% Ag Np's. The extracts were incorporated into the PVA + IA or PVA + Ch solution under continuous stirring for 30 min at room temperature (21°C). Then, the process continued with casting the bioactive film solutions (15 mL) into 90 mm polystyrene Petri dishes, and they were left for solidification at room temperature (21°C) for 48 h. The solidified biofilms were peeled off and subjected to several investigations (e.g., physical measurements, water vapor permeability, etc.).

### 2.4 Storage Test

The blueberries used in this study were sourced from a local market from untreated, ecologically certified agriculture. Only blueberries with optimal appearance, physical integrity, and no signs of mold were selected. They were carefully cleaned and dried with sterile tissue paper after thorough washing with tap water, and stored at 4°C. The six bioactive film formulations (PVA, PVA + Lp + Np, PVA + IA, PVA + IA + Lp + Np, PVA + Ch, PVA + Ch + Lp + Np) were applied as bioactive packaging for storage testing. Uncoated blueberries were also included for comparative shelf‐life analysis. Spoilage was monitored by visual assessment (discoloration, mold growth, shriveling) and microbial analysis (total viable counts and yeast/mold counts). The fruits were weighed using an analytical scale (Kern ALJ 220‐5DNM, Balingen, Germany) with a precision of 0.0001 g, and photographed daily to monitor weight loss and detect any signs of degradation.

#### 2.4.1 Total Number of Germs and Total Number of Yeasts and Molds

A nutrient agar medium was used to determine the total germ count. Samples (3 g) were suspended in 27 mL saline, with two dilutions for day 0 and three for day 7. The pour plate method involved adding 1 mL of each dilution to Petri dishes with 15 mL agar, mixing, and incubating at 30°C for 3 days. Dichloran Rose‐Bengal Chloramphenicol (DRBC) agar was used for yeast and mold count. Samples (3 g) were suspended in 27 mL saline, with two dilutions for both days. The spread plate method applied 0.1 mL of dilution onto agar using a Drigalski spatula. Plates were incubated at 25°C for 5 days (Mitrea et al. 2024). All analyses were done in duplicate.

### 2.5 Antioxidant Activities

#### 2.5.1 2′‐Azino‐Bis (3‐Ethlylbenzothiazoline‐6‐Sulfonic Acid) (ABTS) Radical Cation‐Decolorization Assay

The ABTS radical scavenging activity was assessed as in Nemes et al. (2024). ABTS + solution (7 mM) was mixed with 2.45 mM potassium persulfate and incubated in the dark for 16 h. The working solution (170 μL, 0.70 ± 0.02 AU at 734 nm) was mixed with 20 μL of the sample. After 6 min of incubation, absorbance was measured at 734 nm, and inhibition (%) was calculated as [1 − (sample absorbance/blank absorbance)] × 100 (Nemes et al. 2024). The results are expressed as μmol of Trolox equivalents/100 g of sample.

#### 2.5.2 Cupric Reducing Antioxidant Capacity (CUPRAC)

The CUPRAC method determines the total antioxidant activity of the chosen biofilms. This method presents some compatibility with the DPPH and ABTS methods. This mechanism is due to the redox chemistry of copper (II), developing faster kinetics (Apak et al. 2004). The CUPRAC method was performed according to (Haque and Canete 2018; Hidayat et al. 2019) with slight modifications. The CUPRAC reagent (pH 7) was made by combining equal volumes of 0.01 M CuCl_2_*2H_2_O, 1 M sodium acetate, and 0.0075 M neocuproine. In our modified protocol, 170 μL of the CUPRAC reagent was mixed with 30 μL of the film extract (instead of the typical larger volumes used in standard methods) to suit microplate‐based analysis. The resulting reagent, which is pale blue, reacts with the antioxidants to produce a yellow hue, exhibiting a maximum absorbance at 450 nm in the ultraviolet spectrum. The reagent and sample react for 30 min in the dark before the spectrophotometric reading using a microplate reader (Biotex, USA). The reagent was freshly produced before every experiment. The readings were conducted in triplicate. The antioxidant activity was calculated as a percentage and reported to the blank.

### 2.6 Antimicrobial Activity of Biofilm Solutions

To determine the Minimum Inhibitory Concentration (MIC), the following bacterial and yeast strains were evaluated: *Staphylococcus aureus* ATCC 29213, *Staphylococcus aureus* ATCC 25923, *Escherichia coli* ATCC 25922, *Salmonella enterica* (typhimurium) ATCC 14028, *Candida parapsilosis* ATCC 22019, and *Listeria monocytogenes* DSMZ 115292. Each strain was initially cultured in 10 mL of the appropriate sterile broth medium: tryptic soy broth (TSB) for *E. coli* , *C. parapsilosis* , *S. aureus* , and *P. aeruginosa* ; nutrient broth (NB) for *S. enterica* and tryptic soy yeast extract (TSYE) for *L. monocytogenes* . The cultures were activated by incubating the broth tubes at 37°C for 24 h, while the *Candida* strains were incubated at 30°C for 24 h. The purity of each inoculum was confirmed by microscopic examination of Gram‐stained smears. After incubation, the spread plate method ensured proper colony distribution. Bacterial morphology was verified via optical microscopy. Colonies were transferred to sterile saline (8.5 g/L NaCl) and adjusted to 0.5 McFarland standard (~1.5 × 10^8^ CFU/mL) for MIC testing. (Teleky et al. 2022).

MIC was determined using a resazurin‐based microtiter plate assay. In a 96‐well plate, 100 μL of sterile medium and 100 μL of the sample were added, followed by serial two‐fold dilutions. Each well received 10 μL inoculum (1.5 × 10^5^ CFU/mL for bacteria, 10^6^ cells/mL for yeast). Gentamicin (0.04 mg/mL) and Ketoconazole (1 mg/mL) were positive controls. Plates were incubated at 37°C (bacteria) or 30°C (fungi) for 20 to 22 h. After adding 20 μL of 0.2 mg/mL resazurin, incubation continued for 2 h at 37°C. MIC was recorded as the lowest concentration preventing a blue‐to‐pink color change. Tests were performed in duplicate.

### 2.7 Antibiofilm Activity of Bioactive Film Solutions

The following strains were tested: *Staphylococcus aureus* ATCC 29213, *Pseudomonas aeruginosa* ATCC 27853, *Escherichia coli* ATCC 25922, *Staphylococcus epidermidis* ATCC 12228, *Listeria monocytogenes* DSMZ 115292, and *Salmonella enterica* ATCC 14028. The strains were grown in sterile Nutrient Broth (NB) or Muller Hinton Broth (MHB). The tubes containing tested strains were incubated at 37°C for 24 h. After incubation, a loopful of inoculum was transferred to an agar growth medium, and plates were incubated at 37°C for an additional 24 h. Bacterial morphology was verified using a Nikon ECLIPSE Ci‐L optical microscope.

Biofilm formation started by cultivating bacterial strains overnight on agar. In a sterile microplate, 100 μL culture medium and 100 μL sample were mixed, followed by serial two‐fold dilutions. After 12 dilutions, bacterial suspensions adjusted to McFarland 0.5 (10^8^ CFU/mL) were added (10 μL per well). Plates were incubated at 37°C for 24 h. Planktonic cells were removed by inverting and washing the plates twice with sterile water. Biofilms were stained with 0.1% crystal violet (150 μL) for 20 min at room temperature. Excess stain was rinsed off, and plates were dried inverted for several hours or overnight (Stefanescu et al. 2024). Biofilm quantification involved solubilizing crystal violet with 150 μL of 30% acetic acid per well, followed by 20 min of incubation at room temperature. The solution was transferred to a new microplate, and absorbance was measured at 550 nm using a BioTek plate reader (Winuschi, VT, USA). Biofilm inhibition (%) was then calculated:

(1) $$\text{Percentage}\;\left(\%\right)\;\text{inhibition}=\frac{\left({\mathrm{OD}}_{\text{Negative control}}-{\mathrm{OD}}_{\text{Sample}}\right)\times 100}{{\mathrm{OD}}_{\text{Negative control}}}$$

### 2.8 Rheological Analyses

The flow behavior of the biofilm solutions was analyzed using a modular compact rheometer, Anton Paar MCR 72 (Anton Paar, Graz, Austria), equipped with a Peltier‐controlled plate‐to‐plate system (P‐PTD 200/Air), allowing precise temperature regulation between 5°C and 150°C. Approximately 3 mL of each sample was placed between the plates, with the upper plate featuring a smooth, parallel geometry (50 mm diameter) and the lower plate maintained at 25°C, with a gap of 1 mm (Mitrea et al. 2022). The excess sample was removed before testing, and the samples were equilibrated for 5 min to ensure thermal stability before measurements, performed in duplicate with a shear rate increasing linearly from 5 to 300 s^−1^.

### 2.9 Bioactive Film Characterization

#### 2.9.1 Physical Characterization

The thickness of the films (in μm) was measured at five random points using a digital caliper (Lumytools LT15240, Suceava, Romania), with readings taken in millimeters. Each measurement was performed in triplicate, and the final thickness was calculated as the average of all measurements (*n* = 10). The diameter (in mm) was measured using the same digital caliper at three random points, and the average was calculated from duplicate measurements (*n* = 6).

The mass of the solid films was determined using an analytical scale (Kern ALJ 220‐5DNM, Germany) with a precision of 0.0001 g, and the final mass was averaged from two measurements. The density of the films (g/cm^3^) was calculated using the following formula:
(2)

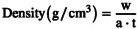

where *w* is the weight of the sample (g), a is the area (cm^2^), and t is the thickness (cm). The final density was calculated using the mean values from two thickness measurements.

#### 2.9.2 Water Vapor Transmission Rate

The water vapor transmission rate (WVTR) was measured following the ASTM E96/E96M‐10 standard. Films were securely placed over permeability cups (VF2200, TQC Sheen, Molenbaan, The Netherlands), each containing approximately 7.5 g of anhydrous CaCl_2_ (0% relative humidity), with a 6 mm air gap between the film and the desiccant. A 10 cm^2^ section of the film was exposed to water vapor. The cups were placed in a desiccator pre‐equilibrated with a saturated NaCl solution at 25°C ± 1°C (75% RH). The cups were weighed five times over a 24‐h period, and a linear regression analysis of weight gain over time was performed. The WVTR was calculated using the formula:

(3) $$\text{WVTR}=\frac{S}{A}$$

where *S* represents the slope of the weight gain curve (g/h) and *A* is the area of the cup mouth (m^2^). All measurements were performed in duplicate (Mitrea et al. 2024).

#### 2.9.3 Water Solubility Test

The biofilms were subjected to a water solubility test based on an adapted protocol with minor modifications (Saputri et al. 2018). Bioactive film samples (2 × 2 cm) were dried in a desiccator with anhydrous calcium chloride for 5 days. The dried biofilm pieces (B0) and Whatman paper no. 1 (W0) with a diameter of 14.5 cm were weighed to the nearest 0.0001 g. The samples were placed in beakers containing 50 mL of double‐distilled water and stirred at 300 rpm for 30 min at 30°C. After stirring, the biofilm solutions were filtered through the Whatman papers, which were subsequently dried at 50°C for 24 h. The dried filter papers were cooled in a desiccator and reweighed (W1). Each measurement was performed in duplicate, and the percentage of total soluble matter, expressed as solubility (%), was calculated using the formula (4). Results are reported as mean ± standard deviation (SD).

(4) $$\text{Solubility}\;\left(\%\right)=\left[B_{0}-\left(W_{1}-W_{0}\right)\right]/B_{0}\times 100$$

where B0 is the weight of the bioactive films (in grams), W0 is the initial weight of the Whatman paper, and W1 is the weight of the filter paper after drying.

#### 2.9.4 Determination of the Swelling Index at Different pH

To evaluate the swelling capacity of the edible biofilm, the swelling index was employed (Choudhary et al. 2023). Initially, each film's dry weight (*Wd*) was recorded. The dry film samples were then immersed in distilled water with varying pH levels of 4, 7, and 9 for 24 h to assess their swelling behavior. After immersion, excess surface water was carefully blotted with filter paper. The films were removed and reweighed periodically until a constant weight (*Ws*) was reached. The swelling index, or water uptake, was calculated using Equation (5). Each pH condition was tested in triplicate, and results are expressed as mean ± SD.

(5) $$\text{Swelling index}\;\left(\%\right)=\frac{\mathrm{Ws}-\mathrm{Wd}}{\mathrm{Wd}}\times 100$$

#### 2.9.5 Color Properties

The color properties of each film were assessed in five randomly chosen areas using a portable spectrophotometer (DS‐700D; China). Following the outlined methodology (B.E. Teleky et al. 2025), we measured the parameters L* (lightness), a* (redness or greenness), and b* (yellowness or blueness). The films' L*, a*, and b* values on a standard whiteboard were recorded as L* = 99.47, a* = −0.09, and b* = 0.10. To quantify the differences in film color (ΔE), we applied the following Equation (6):

(6) $$\Delta E^{*}=\sqrt{\left(L_{2}^{*}+L_{1}^{*}\right)+\left(a_{2}^{*}+a_{1}^{*}\right)+\left(b-b_{1}^{*}\right)\;}$$

#### 2.9.6 Moisture Content

The film's moisture content was determined by measuring the sample's weight loss after drying at 105°C until a constant weight was achieved.

#### 2.9.7 Determination of Biodegradability

The degradation behavior of the bioactive films was assessed through weight loss during soil burial (Choudhary et al. 2023). Samples (2 × 2 cm) were buried 2 cm deep and spaced 3 cm apart, and their initial weight (Wi) was measured. At regular intervals of 7 days (for 8 weeks), the samples were weighed using an analytical balance with a precision of 0.0001 g, both before and after the degradation period. The percentage of weight loss was determined using the Equation (7):

(7) $$\mathrm{Bs}=\frac{\mathrm{Wi}-\mathrm{Wf}}{\mathrm{Wf}}\times 100$$

where *Wi* and *Wf* are the initial weight and final weight of the sample after a 7‐day period.

### 2.10 Statistical Analyses

All experiments were performed in duplicate or triplicate, with results expressed as mean values ± standard deviation (SD). Data normality was evaluated using the Shapiro–Wilk test, with a *p‐*value of less than 0.05 considered statistically significant. The Brown‐Forsythe test was used to assess variance equality, followed by one‐way ANOVA and Tukey's post hoc multiple comparisons to identify significant differences between PVA and enriched bioactive films. After confirming normality, two‐way ANOVA and Tukey's post hoc tests were applied for other experiments. Statistical analyses were conducted using GraphPad Prism (Version 9.3.0, GraphPad Software Inc., San Diego, CA, USA).

## 3 Results and Discussion

### 3.1 Coating Solution Preparation, Characterization, and Application

This study developed and evaluated bioactive films for sustainable food packaging, focusing on blueberries through immersion. AgNps and Lp were incorporated to enhance antioxidant and antimicrobial properties, preserving fruit quality. Tests showed reduced weight loss, microbial growth, and spoilage. The films addressed challenges like softening and shrinkage, thus effectively extending shelf life.

#### 3.1.1 Characterization of AgNp

Before incorporating AgNp into the bioactive film solution, the surface charge and colloidal stability were determined by size, zeta potential, and polydispersity index (PdI) (Table 1). The particle size of AgNp confirmed the nanoscale range. For instance, Np's used in biofilm matrices typically varied between 30 and 50 nm, as Vitale et al. (2022) suggested; such sizes ensure effective dispersion within the polymer matrix, enhancing mechanical and barrier properties (Vitale et al. 2022). Additionally, the ζ‐potential of −29.2 ± 1.41 mV showed good colloidal stability, a property critical for maintaining the functional uniformity of Np's.

**TABLE 1:** Size, zeta potential, and polydispersity index of AgNP.

	Hydrodynamic diameter (nm)	ζ‐potential (mV)	Polydispersity index
AgNp	44 ± 2	−29.2 ± 1.41	0.28 ± 0.02

The relatively low PdI enhanced the homogeneity of Np‐infused films. The uniform dispersion of Np‐Ag within PVA and Ch matrices can potentially influence functional properties (Rao et al. 2024). Germini and Peltonen (2021) results suggested that low PdI values correlate with improved film characteristics such as flexibility and uniformity (Germini and Peltonen 2021).

**FIGURE 1:**
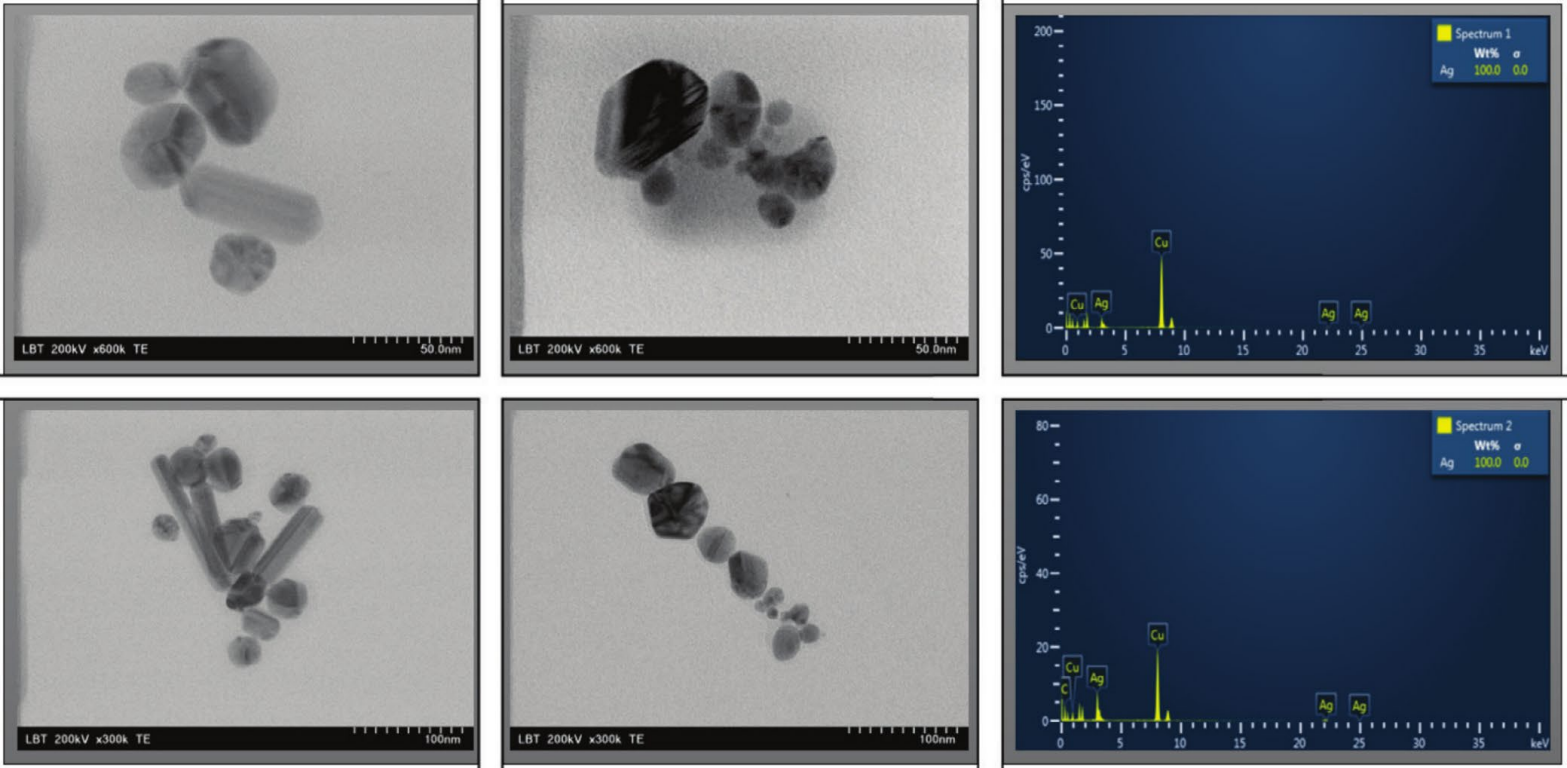
TEM images and EDTX maps of the synthesized silver nanoparticles.

To support the claimed DLS results on surface charge and colloidal stability, the AgNp with different synthesis methods was analyzed by TEM (Figure 1). The TEM analysis of AgNp exhibited a uniform morphology, primarily spherical with some polyhedral structures, indicating slight heterogeneity in shape. The particle sizes align with the previously discussed hydrodynamic diameter values (~44 nm). The images reveal a relatively uniform particle size distribution, with minimal aggregation observed, and the clear boundaries of the Np's indicate a well‐defined crystallinity.

The EDX spectra further validate the elemental composition, indicating the presence of Ag as the primary element. Also, Ag's quantitative weight percentage (Wt%) confirmed the elemental purity, revealing significant Ag depletion under electron beam exposure. During the synthesis process, the heating of the AgNO_3_ solution and the addition of sodium citrate facilitate the reduction of Ag^+^ ions to elemental silver (Ag^0^) (La Spina et al. 2020; Tessema et al. 2023). Sodium citrate provides electrons, enhancing the nucleation and development of AgNp.

#### 3.1.2 Phenolic Profile of Lemon Peel Extract

The phenolic profile of Lp extract (Table 2) revealed a total phenolic content of 7396.77 ± 5.40 μg/mL, with increased amounts of flavanones such as eriodictyol (3273.23 ± 27.26 μg/mL) and eriocitrin (1086.28 ± 6.31 μg/mL), alongside flavones like diosmetin‐glucoside (380.13 ± 16.43 μg/mL). This composition underscores the bioactive potential of Lp, especially due to its flavonoid content, which is widely recognized for its antioxidant and functional properties.

**TABLE 2:** Identification and quantification of phenolic compounds in lemon peel extract (μg/mL).

Peak Nr.	R_t_ (min)	[M + H]^+^ (m/z)	UV λ_max_ (nm)	Phenolic compounds	Subclass	Lemon peel
1	12.99	595, 287	274, 341	Isosakuranetin‐neohesperidoside (Poncirin)	Flavanone	975.175 ± 0.632
2	13.30	595, 287	274, 341	Isosakuranetin‐rutinoside (Didymin)	Flavanone	475.889 ± 1.136
3	13.56	625, 301	270, 342	Diosmetin‐diglucoside	Flavone	161.711 ± 0.206
4	15.40	597, 289	280, 334	Eriodictyol‐rutinoside (Eriocitrin)	Flavanone	1086.276 ± 6.310
5	16.23	463, 301	270, 342	Diosmetin‐glucoside	Flavone	380.134 ± 16.430
6	17.70	463	260, 360	Luteolin‐glucuronide	Flavone	170.962 ± 1.027
7	20.81	289	280	Eriodictyol	Flavanone	3273.229 ± 27.259
8	23.18	303	280	Hesperetin	Flavanone	873.399 ± 1.199
				Total phenolics		7396.772 ± 5.401

*Note:* Mean ± SD (*n* = 3).

Abbreviation: Rt, retention time.

A similar study by Athanasiadis et al. (2024) reported a TPC of 526.32 mg GAE/L using cloud point extraction (CPE), with eriocitrin (159.43 mg/L) and hesperidin (135.21 mg/L) as dominant polyphenols. This similarity emphasizes the efficiency of ultrasound and CPE methods for extracting bioactive compounds (Athanasiadis et al. 2024).

Lp extract is a rich source of bioactive compounds, particularly flavonoids like eriocitrin, hesperidin, and diosmin. These compounds exhibit potent antioxidant, anti‐inflammatory, and antimicrobial properties. Eriocitrin is important in combating oxidative stress and supporting metabolic health, while hesperidin is linked to improved cardiovascular function and cognitive protection (Matsuzaki et al. 2022). Additionally, Lp extract helps regulate blood glucose levels and promotes healthy gut microbiota, making it a promising ingredient for functional foods and dietary supplements. Moreover, it can be incorporated into biodegradable films and eco‐friendly materials, enhancing their functionality for active food packaging (Fakraoui et al. 2024). Overall, Lp extract is a versatile and health‐promoting resource with environmental benefits.

#### 3.1.3 Antioxidant Activities

**FIGURE 2:**
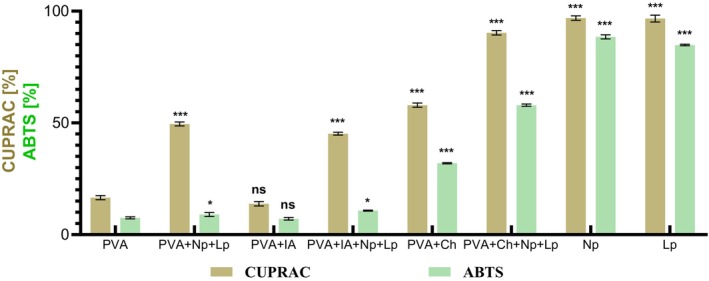
The antioxidant activity of PVA‐based bioactive films was evaluated using the ABTS and CUPRAC assays. The results are expressed as mean values ± SD (*n* = 3). A two‐way ANOVA followed by Tukey's multiple comparisons test was performed, with *p*‐values indicated as follows: ***p < 0.001, *p < 0.05, and ns (not significant) for *p* > 0.05.

The ABTS assay results provide valuable insights into the antioxidant capacity of the tested samples. Among the evaluated formulations, the Lp extract exhibited high antioxidant activity, achieving an inhibition percentage of 84.86% (Figure 2). This result highlights the free radical scavenging ability of Lp, which is attributed to its high content of bioactive compounds such as polyphenols, flavonoids, limonoids, and vitamin C (Gao et al. 2020; Saleem et al. 2023). Incorporating Lp extract into PVA‐based bioactive films significantly enhances their antioxidant properties, making them promising for applications where oxidative stability is essential, such as in food packaging (Sahraee et al. 2019) or biomedical fields (Liu et al. 2024). The inclusion of Np's brought notable changes in the antioxidant activity of the bioactive film formulations. For instance, the sample PVA + Ch + Np + Lp demonstrated an inhibition percentage of 57.94%, significantly (***p < 0.001) improving compared to the PVA bioactive film alone (7.53%). This suggests a synergistic effect between the Np's and the bioactive compounds in the Lp extract, enhancing the overall antioxidant performance (Khane et al. 2022). Furthermore, samples combining multiple components (e.g., PVA + Ch + Np and PVA + IA + Np + Lp) showed moderate antioxidant activities of 33.86% and 10.74%, respectively, indicating that the specific interactions between the ingredients play a critical role in determining the overall performance. The addition of Np's and a stabilizing effect, particularly in complex formulations, extending the utility of the biofilms in oxidative environments.

Using the CUPRAC method, the antioxidant activity of the biofilms increased in the following order: PVA + IA < PVA < PVA + IA + Np + Lp < PVA + IA + Np < PVA + Np + Lp < PVA + Np < PVA + Ch < PVA + Ch + Np < PVA + Ch + Np + Lp < Lp < Np. The difference between the methods' results may occur due to each radical's different modes of action. For example, the CUPRAC reagent is very selective; hence, not all potential antioxidants are oxidized. Figure 2 shows that the best antioxidant activity was measured by PVA + Ch + Np + Lp biofilm (90.36%).

Integrating PVA with Ch, Np's, and Lp inside a bioactive film produces a synergistic effect, amplifying the antioxidant activity beyond the capabilities of each component alone. PVA alone exhibits negligible antioxidant activity (15.97%), although it functions as a matrix with high potential to integrate other active compounds (Hajji et al. 2016). Enriching PVA bioactive films with Np's and Lp significantly increased (*p < 0.05) the antioxidant activity against the simple biofilm (PVA + Np + Lp 49.52%). Ch has antioxidant properties because its amino groups can donate hydrogen atoms to neutralize free radicals. Its positive charge also helps it interact with negatively charged reactive oxygen species (ROS), which improves its ability to remove these harmful radicals (Hromis et al. 2017). Its film‐forming ability also supports the stable dispersion of Np's and Lp, further amplifying their efficacy (Mehdizadeh et al. 2022). Previous reports by Nair et al. (2019) also revealed enhanced scavenging activity in PVA‐encapsulated citrus peel (Nair et al. 2019). The Ch samples register the highest antioxidant activity through the tested biofilms (PVA + Ch + Np 61.41%, PVA + Ch + Np + Lp 90.36%), comparable with Np's and Lp alone.

Conversely, IA is known for its mild antioxidant effects when alone (Figure 2). However, when added to the PVA matrix, it consistently has the opposite effect (the antioxidant activity decreases by ≃4% when IA is used). This could be due to its role in cellular metabolism as a product of the Krebs cycle. Under certain oxidative conditions, it may act as a pro‐oxidant by affecting redox‐sensitive signaling pathways or producing ROS intermediates (Fedotcheva and Beloborodova 2022). Results showed that IA negatively impacts antioxidant activity in the PVA biofilms (PVA + IA 13.82%).

#### 3.1.4 Antibiofilm and Antibacterial Activity of Bioactive Film Solutions

**FIGURE 3:**
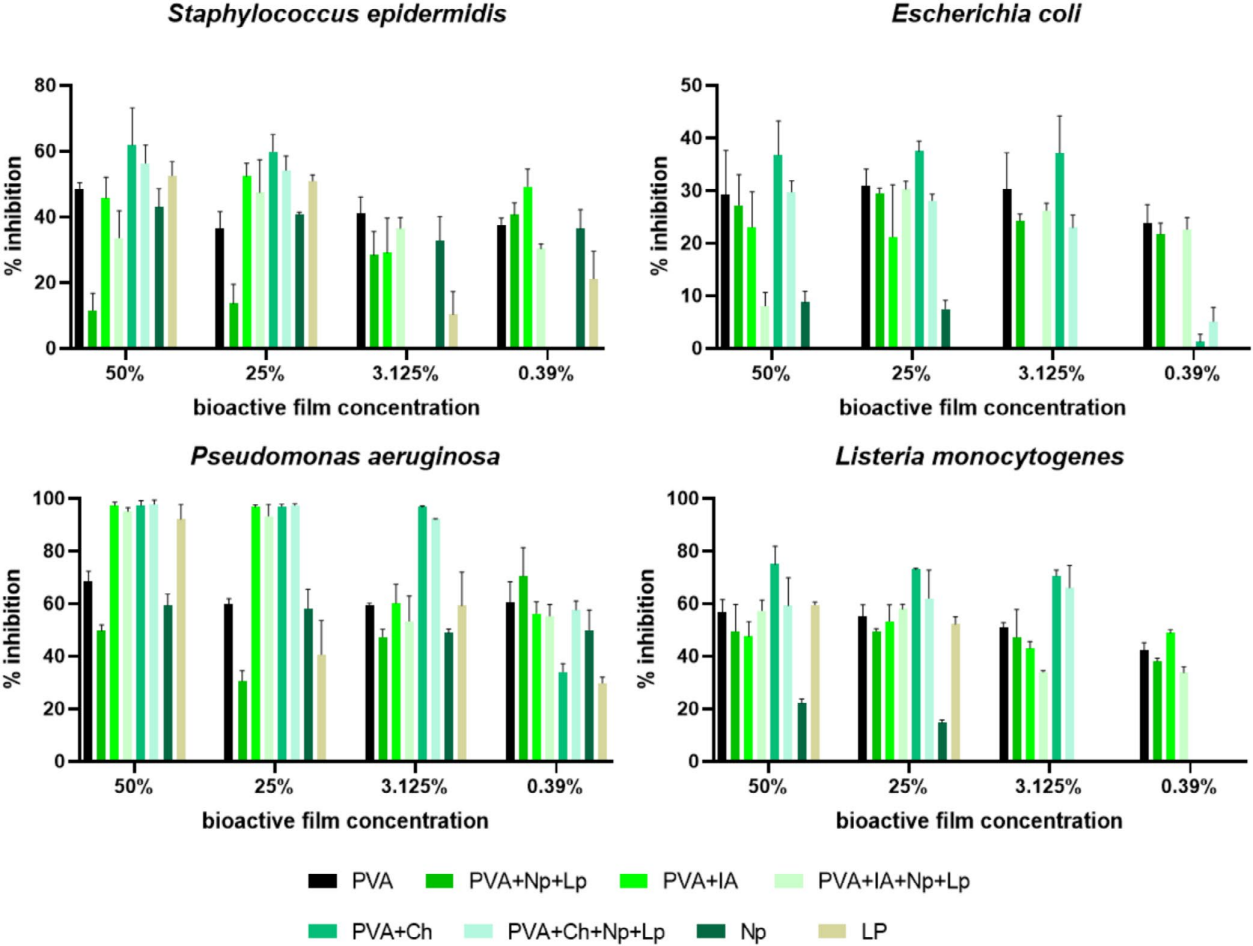
The biofilm inhibition efficiency of different formulations against four bacterial strains at varying concentrations. Biofilm (BF) inhibition was categorized on a scale from 0% to 100%. Values below 0% were recorded as 0% BF inhibition. Those falling between 0% and 50% indicated weak anti‐biofilm activity, and values exceeding 50% denoted effective biofilm inhibition. Any values surpassing 100% were reported as 100% BF inhibition.

The results (Figure 3) demonstrate a clear concentration‐dependent antibiofilm effect, with higher concentrations of bioactive film generally resulting in more effective inhibition, mainly when more complex and synergistic combinations of components were utilized.

Formulations containing various combinations of PVA, Np, Lp, and IA consistently exhibited superior antibiofilm activity to formulations containing single components. For *S. epidermidis* , significant inhibition was observed at the highest bioactive film concentrations of 50% and 25%. Among the tested formulations, those containing PVA + Np + Lp, PVA + IA + Np + Lp, and PVA + Ch proved to be the most effective, achieving higher inhibition percentages than single components or simpler combinations. When combined, these agents produce a synergistic effect: chitosan improves the dispersion and stabilization of AgNp's in the polymer matrix, enhancing their bioavailability and prolonging antimicrobial and antibiofilm activity. However, as the bioactive film concentration decreased to 3.125% and 0.39%, the inhibition percentages declined substantially. *E. coli* showed a similar concentration‐dependent pattern, although the overall inhibition percentages were less pronounced than *S. epidermidis* . At 50% and 25% bioactive film concentrations, combinations such as PVA + Ch + Np + Lp and PVA + Ch demonstrated the highest inhibition rates, reinforcing the synergistic impact of using multiple active components. However, at lower bioactive film concentrations, such as 3.125% and 0.39%, the inhibitory effect was significantly reduced, indicating that *E. coli* biofilms may be more resilient to the tested formulations at lower densities or that the formulations require optimization to sustain activity across a broader concentration range.

In the case of *P. aeruginosa* , the tested formulations displayed the highest levels of antibiofilm activity among all the studied strains. Nearly complete suppression of biofilm formation was achieved at bioactive film concentrations of 50% and 25%, mainly with formulations containing PVA + Np + Lp, PVA + IA + Np + Lp, and PVA + Ch + Np + Lp. Even at a reduced bioactive film concentration of 3.125%, these formulations maintained notable inhibition, highlighting the susceptibility of *P. aeruginosa* to the active components. This strain's biofilm structure and metabolic activity may render it more vulnerable to the synergistic effects of the tested formulations, even at lower concentrations. At the lowest concentration of 0.39%, the inhibition declined but remained measurable, further emphasizing the robustness of these formulations against *P. aeruginosa* . The inhibition trends for *L. monocytogenes* resembled those observed for *S. epidermidis* . In combination, formulation involving IA and Np provided the most significant levels of biofilm suppression, particularly at 50% and 25% bioactive film concentrations. At these levels, combinations such as PVA + IA + Np + Lp demonstrated a pronounced inhibitory effect, likely due to the enhanced activity resulting from the interaction of these components. In contrast, single components such as PVA or Np alone showed limited activity, suggesting that synergistic interactions between the compounds are critical for achieving effective biofilm inhibition. As the bioactive film concentration decreased to 3.125% and 0.39%, the inhibitory effects of the formulations declined, indicating a concentration‐dependent efficacy similar to that observed for the other strains. According to the literature (Chegini et al. 2024), when bacteria come into contact with AgNp's, the Np's attach to the bacterial cell wall and undergo oxidation. This process releases a high concentration of Ag^+^ ions at the interface between the Np's and the bacterial cells. Consequently, silver ions (Ag^+^) can induce oxidative stress and cause DNA damage in bacterial cells. Extracellular polymeric substances, primarily composed of proteins, polysaccharides, lipids, and nucleic acids, play a vital role in the adhesion and biofilm formation of *L. monocytogenes* . According to Peng et al. (2024), the levels of protein, polysaccharides, and eDNA in the groups treated with citrus peel essential oil were significantly lower (*p* < 0.05) compared to the untreated control group (Peng et al. 2024). In the 2% (*v/v*) treated group, the EPS protein, polysaccharide, and eDNA content decreased by 47.77%, 54.74%, and 63.74%, respectively.

Similar results were obtained by Thaya et al. (2018), who demonstrated that Ch‐alginate microspheres effectively inhibited bacterial biofilm formation in *S. aureus* , *E. faecalis* , *P. aeruginosa* , and *P. vulgaris* after a single treatment with 40 μg. This antibiofilm activity is attributed mainly to Ch's positive charge, enabling it to interact with the negatively charged bacterial cell membrane, disrupting membrane permeability and affecting its structural and functional integrity (Thaya et al. 2018). Also, Al‐Fawares et al. (2024) developed Ch Np's cross‐linked with sodium tripolyphosphate and coated with polyacrylic acid (PAA), significantly inhibiting biofilm formation at a 0.5 mg/mL concentration. In situ optical microscopy revealed that Ch‐PAA Np's effectively prevented biofilm formation in *Campylobacter jejuni* , *P. aeruginosa* , and *E. coli* after a single treatment with 40 μg (Al‐Fawares et al. 2024).

The results presented in Table 3 indicate that formulations containing Ch (PVA + Ch and PVA + Ch + Np + Lp) exhibited inhibitory effects, with MICs lower than those of other formulations. This underscores the crucial role of Ch in antimicrobial activity. For Gram‐negative bacteria, both PVA + Ch and PVA + Ch + Np + Lp displayed potent inhibitory effects, with MICs as low as 0.78% for PVA + Ch, *S. enterica* ATCC 14028. This high susceptibility is likely due to the ability of Ch to disrupt the outer membrane and cell wall integrity, which are characteristic of Gram‐negative bacteria (Sun et al. 2022). In Gram‐positive bacteria, while the formulations remained effective, slightly higher MIC values were observed, with PVA + Ch achieving a 0.78% bioactive film concentration with greater efficacy than PVA + Ch + Np + Lp. The yeast *C. parapsilosis* demonstrated higher resistance to the tested formulations than bacterial strains, with inhibition achieved only at higher concentrations (12.5%) of PVA + Ch and PVA + Ch + Np + Lp. This observation highlights the greater resilience of fungal cells to antimicrobial agents, likely due to their unique cell wall composition, which includes chitin and glucans that provide structural strength and reduce permeability (Cortes et al. 2019). While some evidence suggests Gram‐negative bacteria are more susceptible to Ch, others indicate that Gram‐positive bacteria, due to their teichoic acids, are more sensitive. However, the thick cell wall of Gram‐positive bacteria may act as a barrier, although small Ch molecules can penetrate and disrupt essential cellular processes like DNA and RNA synthesis. Ch's antibacterial effect is determined by its molecular size and structural properties (Ke et al. 2021).

**TABLE 3:** The results of determining the minimum inhibitory concentration (MIC) (% bioactive film solution) of the bioactive film solutions.

Sample (% bioactive film solution)	Gram‐negative bacteria (−)		Gram‐positive bacteria (+)			Yeast
	*E. coli* ATCC 25922	*S. enterica* ATCC 14028	*S. aureus* ATCC 25923	*L. monocytogenes* DSMZ 115292	*S. aureus* ATCC 29213	*C. parapsilosis* ATCC 22019
PVA	n.b.	n.b.	n.b.	n.b.	n.b.	n.b.
PVA + Np + Lp	n.b.	n.b.	n.b.	n.b.	n.b.	n.b.
PVA + IA	n.b.	n.b.	n.b.	n.b.	n.b.	n.b.
PVA + IA + Np + Lp	n.b.	n.b.	n.b.	n.b.	n.b.	n.b.
PVA + Ch	1.56	0.78	0.39	0.39	3.12	12.5
PVA + Ch + Np + Lp	1.56	1.56	0.78	0.78	3.12	12.5
Np	n.b.	n.b.	n.b.	n.b.	n.b.	n.b.
Lp	n.b.	n.b.	n.b.	n.b.	n.b.	n.b.
Gen.	1.56	0.09	0.02	0.19	0.39	n.b.
KTZ	n.b.	n.b.	n.b.	n.b.	n.b.	25

Abbreviations: Gen., gentamicin solution 0.4 mg/mL; KTZ, ketoconazole solution 1 mg/mL; n.b., no bioactivity.

Similar results were reported by Raphael and Meimandipour (2017), who investigated the antimicrobial activity of Ch film‐forming solutions enriched with essential oils. Their study tested the films against microbial strains such as *Staphylococcus aureus* , *E. coli* , *Enterococcus faecium* , *Lactobacillus rhamnosus* , *Aspergillus niger*, and *Alternaria alternata* . The combination of essential oils and Ch solution demonstrated a bactericidal effect across all tested bacterial strains, with varying degrees of susceptibility. Specifically, the Ch films, the control ones, exhibited a MI of 2.5 μL/mL for *E. coli* and *S. aureus* . This aligns with our findings of MIC values of 1.56 and 0.39 μL/mL, respectively, for Ch‐PVA films. Regarding fungi from the species *Aspergillus* and *Alternaria*, the Ch films showed a MIC exceeding 10 μL/mL (Raphael and Meimandipour 2017).

#### 3.1.5 Rheological Analyses of Biofilm Solutions

**FIGURE 4:**
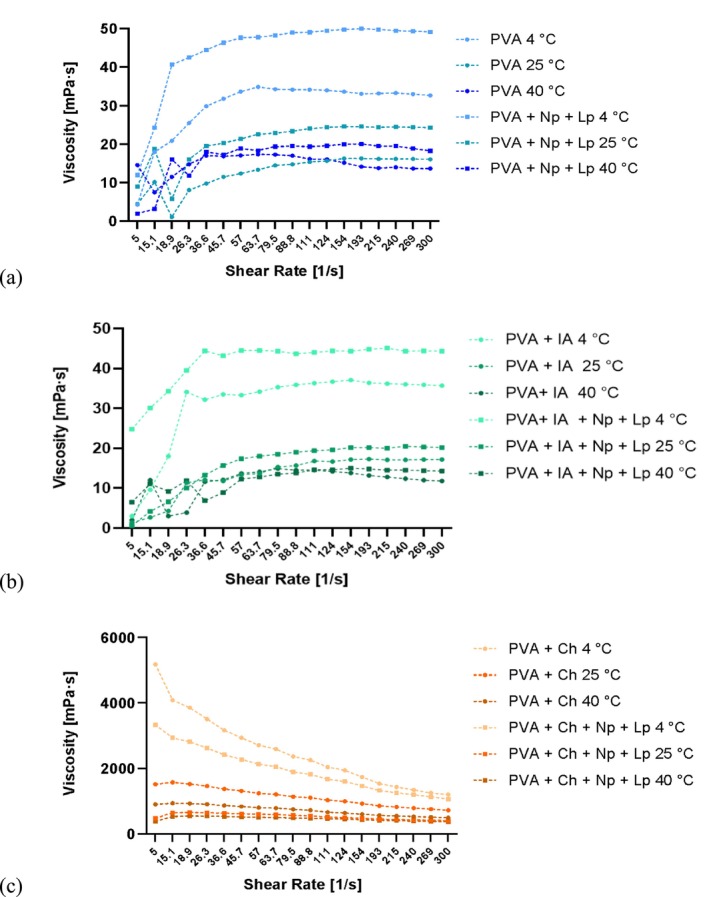
Viscosity‐shear rate relationship for (a) PVA, (b) PVA + IA, and (c) PVA + Ch biofilm solutions, evaluated at three temperatures: 4°C, 25°C, and 40°C.

The semi‐crystalline polymer PVA contains hydroxyl groups (–OH) that form intra‐ and intermolecular hydrogen bonds, significantly affecting its solutions' mechanical and rheological properties. Figure 4a–c illustrates the viscosity‐shear rate relationship for the PVA‐based biopolymer solutions (PVA, PVA + IA, and PVA + Ch) at three different temperatures (4°C, 25°C, and 40°C). The viscosity of pure PVA solutions was the lowest across all conditions (32.7, 16.1, 13.7 mPa·s), reflecting its baseline behavior as a flexible and water‐soluble polymer. Incorporating IA resulted in a slight increase in viscosity due to the presence of carboxylic acid groups (35.7, 17.2, 11.8 mPa·s), which promote stronger intermolecular interactions. PVA with Ch demonstrated the highest viscosity, attributed to robust hydrogen bonding between the PVA and Ch molecules (1205.5, 726.6, 499.8 mPa·s).

Adding AgNp's and Lp extract further increased the viscosity in all formulations. AgNp's act as fillers, restricting the mobility of polymer chains and creating a more structured and rigid network within the solutions. Meanwhile, Lp's bioactive compounds and fibers interact with the polymer matrix, enhancing its rheological properties. The temperature variations revealed that the solutions exhibited the highest viscosity at 4°C due to reduced molecular motion, while increasing temperatures (25°C and 40°C) led to progressively lower viscosities, reflecting greater polymer mobility and network disruption.

PVA and PVA + IA presented shear‐thickening (dilatant) behavior, characterized by increased viscosity with increasing shear rate. In contrast, PVA + Ch solutions displayed non‐Newtonian shear‐thinning (pseudo‐plastic) behavior, where viscosity decreased under higher shear rates. The shear‐thinning property of PVA + Ch is particularly advantageous for film formation, as it facilitates ease of application during processing while maintaining stability at rest. The shear‐thinning properties and temperature‐dependent viscosity reduction were similar to trends observed in alginate films with olive leaf extract, exhibiting thermal sensitivity and additive‐enhanced viscosity. In both studies, incorporating bioactive additives improved solution stability by reinforcing polymer networks and restricting molecular mobility (Mitrea et al. 2024). The viscosity and mechanical properties improvements highlight the suitability of AgNp—and Lp‐enriched PVA‐based biofilms for advanced packaging applications, particularly under diverse environmental conditions.

#### 3.1.6 Blueberry Storage Test

**FIGURE 5:**
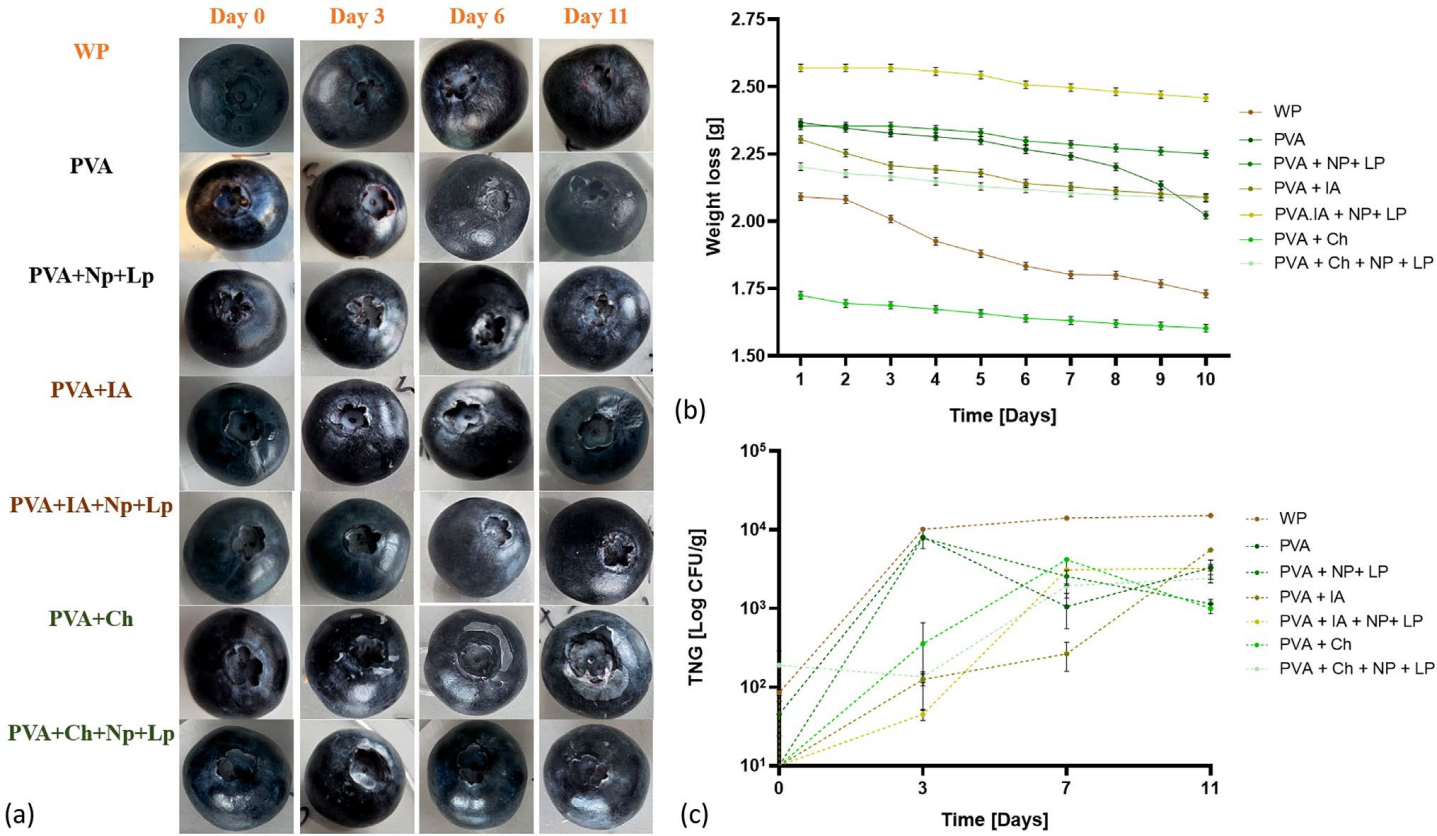
(a) Visual assessment of blueberries on days 0, 3, 6, and 11; (b) Weight loss through 11 days of storage; (c) Total number of germs [TNG] on days 0, 3, 7, and 11.

The effectiveness of synthesized edible biofilms in preserving blueberries was assessed by examining the visual condition of the fruit wrapped by immersion in each biofilm solution. Photographic evidence (Figure 5a) was collected and analyzed over an 11‐day storage period at 4°C. The application of edible coatings has been shown to significantly enhance the postharvest preservation of blueberries by establishing a selective barrier on the fruit's surface, which alters the surface microenvironment and enhances storage quality (Shi et al. 2024). Visual quality is vital for consumer acceptance. Coated blueberries consistently appeared fresher and more appealing throughout the storage duration (4.31%–4.38%), especially with biofilms enhanced with Np and Lp. The biofilm likely provided a protective layer, reducing surface damage and preserving the berries' natural shine and color. In contrast, uncoated blueberries (17.61%) exhibited signs of shriveling and discoloration earlier, reflecting moisture loss and oxidative damage (Davidovic et al. 2021).

Blueberries' weight loss (Figure 5b) indicates moisture loss, which adversely affects freshness. Coated blueberries displayed significantly less weight loss than their uncoated counterparts, likely due to the coating's barrier properties. This barrier minimizes transpiration and evaporation, thus preserving the fruit's internal moisture content. Conversely, the uncoated samples suffered from accelerated dehydration, resulting in more significant weight loss and reduced shelf life. Moreover, the total microbial count (Figure 5c) found on the blueberries further highlighted the efficacy of the coating. The coating likely functioned as an antimicrobial or antioxidant barrier, preventing the proliferation of microorganisms on the fruit's surface (Mannozzi et al. 2017). In contrast, the uncoated blueberries exhibited significantly higher microbial loads, likely due to direct exposure to environmental contaminants and insufficient surface protection. Additionally, the results of the total number of yeasts and molds were 0 in all analyzed samples, suggesting that both coated and uncoated blueberries effectively resisted fungal contamination during storage. This could be attributed to the storage conditions at 4°C, which are known to suppress the growth of yeasts and molds. However, the absence of packaging in the uncoated samples highlights the natural resistance of blueberries to fungal contamination under low‐temperature conditions, suggesting that the primary advantage of the biofilm coatings lies in their ability to reduce weight loss and microbial contamination without introducing additional risks of fungal growth.

### 3.2 Solid Bioactive Film Characterization and Biodegradability Assay

Characterizing solid biofilms and evaluating their biodegradability are crucial for developing sustainable materials for food packaging. These assessments reveal the films' physical, mechanical, and barrier properties, while biodegradability tests ensure environmental compatibility. Together, these analyses highlight the potential of bioactive films to reduce waste, maintain food quality, and align with circular economy principles.

#### 3.2.1 Physical Measurements, Water Vapor Transmission Rate, Swelling Index, and Water Solubility Test

**FIGURE 6:**
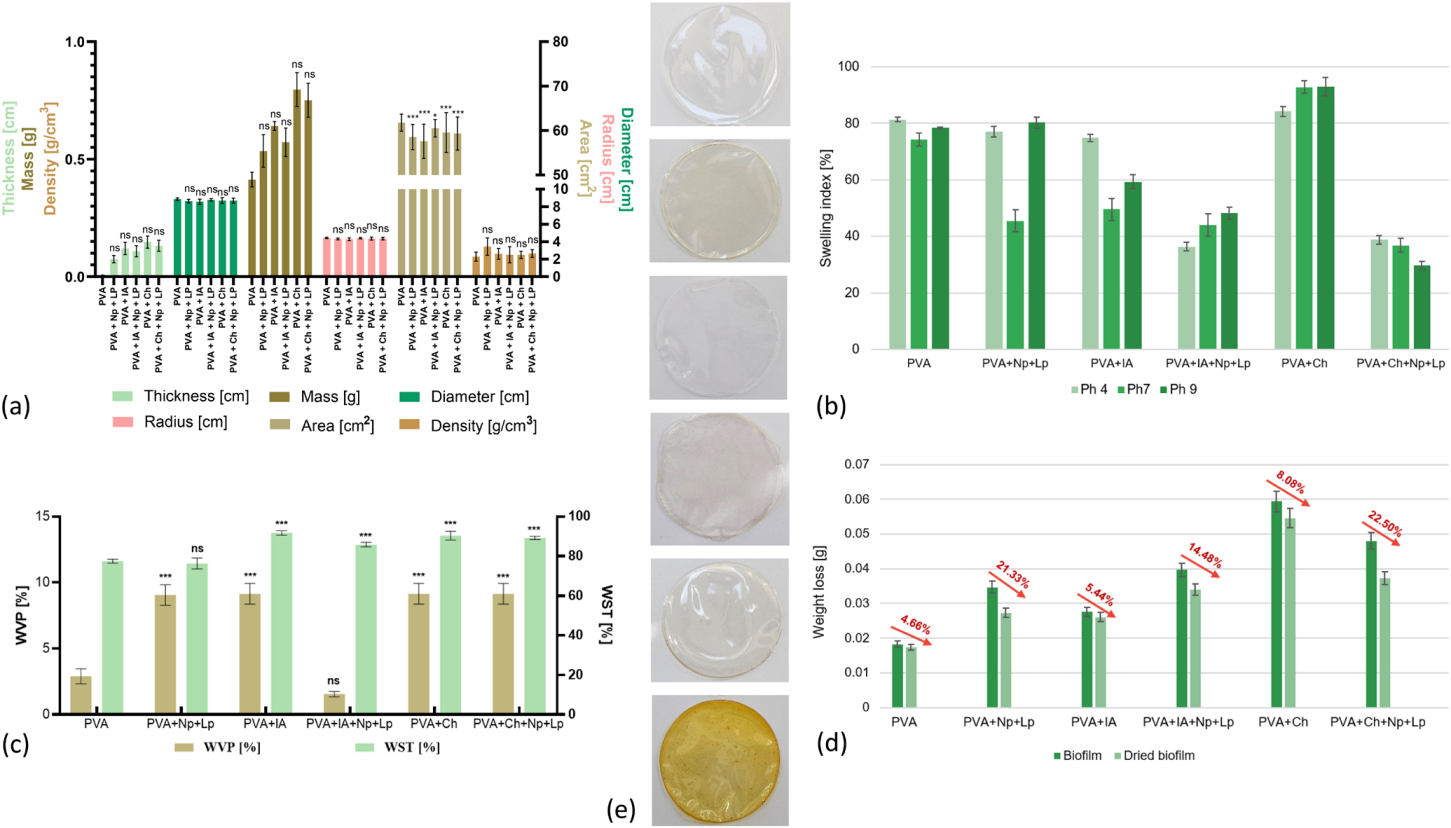
(a) Physical characterization; (b) swelling index at pH 4, pH 7, and pH 9; (c) water vapor permeability (WVP) and water solubility test (WST); (d) weight loss (moisture content); (e) physical appearance of the PVA‐based bioactive films. The results are expressed as mean values ± SD (*n* = 3). A two‐way ANOVA followed by Tukey's multiple comparisons test was performed, with *p*‐values indicated as follows: ***p < 0.001, **p < 0.01, *p < 0.05, and ns (not significant) for *p* > 0.05.

The physical characterization data illustrate the impact of incorporating functional additives into the PVA matrix. PVA + Ch and PVA + Ch + Np + Lp exhibit the highest thickness and density, indicating the reinforcing effects of Ch and Nps (Figure 6a). Ch's molecular structure facilitates strong intermolecular hydrogen bonding with PVA, creating a denser matrix. These results align with studies showing that adding Ch to PVA improves film mechanical properties by enhancing the polymer network integrity (Firdaus et al. 2024). Similarly, PVA + IA + Np + Lp shows a marked increase in thickness and density, reflecting the synergistic effects of IA and Np's, which enhance cross‐linking and reduce polymer mobility. Conversely, the control PVA film demonstrates the lowest values for all physical properties, consistent with its lower structural complexity. Recent work by Jayakumar et al. (2023) also highlights the role of Np's in improving film rigidity and dimensional stability, supporting the observed trends (Jayakumar et al. 2023).

The WVP values of the bioactive films ranged from 1.55% ± 0.20% (PVA + IA + Np + Lp) to 9.15% ± 0.78% (PVA + Ch + Np + Lp). These results are comparable to or higher than those reported in similar PVA‐based biodegradable films, where WVP values typically range between 2.0% and 5.0% under similar conditions (Zomorodian et al. 2023). The improved barrier properties are likely due to increased intermolecular interactions and denser film structure resulting from hydrogen bonding and Np reinforcement.

The swelling index, as observed in Figure 6b, is the pH‐responsiveness of the films. PVA + Ch (between 84.20%–92.95%) and PVA (74.34%–81.30%) exhibit the highest swelling indices across all pH levels. Ch's hydrophilic amino groups and IA's carboxylic acid groups enable significant water uptake under acidic and neutral conditions, as observed in previous studies (Ragab et al. 2024). The reduced swelling at pH 9 for PVA + Ch + Np + Lp of 29.78% reflects deprotonation of functional groups in alkaline environments, reducing water absorption. The moderate swelling observed for PVA + Ch + Np + Lp is attributed to the structural reinforcement provided by Np's, which limits water penetration. In the case of PVA + IA + Np + Lp, the swelling index was significantly lower (***p < 0.001) than observed in the control films (PVA). These findings align with Xue et al. (2024), who demonstrated that incorporating IA into polymer matrices enhances pH sensitivity by introducing carboxylic groups that interact with environmental conditions (Xue et al. 2024).

The WVP and WST data indicate that adding Ch and IA significantly enhances barrier properties (Figure 6c). PVA + Ch and PVA + IA + Np + Lp exhibit the lowest water vapor permeability (***p < 0.001), consistent with forming denser polymer networks that restrict water diffusion. Ch gel‐forming capability and its interactions with PVA hydroxyl groups are key in reducing permeability (Jayakumar et al. 2023).

The WST results also confirm reduced solubility for these formulations, particularly PVA + Ch, which is highly resistant to water dissolution due to the strong intermolecular bonds in its matrix. Firdaus et al. (2024) reported that Ch‐enhanced films demonstrate lower solubility due to their cohesive polymer structure. The control PVA film shows the highest solubility and permeability, reflecting its hydrophilic nature and absence of cross‐linking additives (Firdaus et al. 2024).

The weight loss of the biofilms (Figure 6d), which indicates moisture content, varied significantly across different formulations. PVA biofilms exhibited the lowest weight loss at 4.66%, reflecting their compact structure and limited moisture evaporation. When Np's and Lp were incorporated into the PVA, the weight loss increased to 21.33%. This higher loss is likely due to the hygroscopic nature of these bioactive components. The addition of the IA reduced the weight loss to 5.44%, suggesting that IA enhances the water retention capacity of the films by increasing cross‐linking in the polymer matrix. Similarly, the formulation PVA + IA + Np + Lp showed moderate weight loss of 14.48%, which appears to balance the effects of cross‐linking and the hydrophilic nature of the additional components. In contrast, the formulations PVA + Ch and PVA + Ch + Np + Lp exhibited higher weight loss rates of 8.08% and 22.50%, respectively. This can be attributed to the hydrophilic nature of Ch and its interaction with other bioactive additives, leading to increased moisture absorption and release. The highest weight loss observed in the PVA + Ch + Np + Lp formulation underscores the combined effect of Ch, Nps, and Lp extract on moisture retention, enhancing evaporation rates due to their porous structure. The data indicate that bioactive film formulations with higher bioactive components tend to lose more moisture, which could impact their mechanical strength and barrier properties.

### 3.3 Color Properties

The 𝐿*, a*, and b* color parameters provide insights into bioactive PVA‐based films' lightness and color characteristics, highlighting their potential for food packaging and visual appeal. PVA had an L* value of 35.99 ± 0.63, indicating moderate lightness (Table 4 and Figure 6e). Adding IA, Lp, and Np's increased this value to 38.17 ± 0.11 (*p* ≤ 0.05), suggesting enhanced brightness due to increased light scattering from Np's and better film homogeneity. Conversely, PVA + Ch + Np + Lp showed a lower L* value of 35.17 ± 0.12, attributed to light absorption and scattering influenced by aggregated components. These changes result from specific chemical interactions between the additives. The hydrogen bonding between the hydroxyl groups in PVA and the carboxyl or phenolic groups in IA and Lp likely affects how the polymer chains pack and how pigments are spread out. Additionally, the amino groups in Ch can interact with the negatively charged phenolic compounds from Lp or the surface charges on Np's. This interaction helps stabilize the pigments, contributing to a uniform color. Firdaus et al. (2024) found that adding Lp pectin and plant extracts to Ch films lowered L* values. The a* parameter reflects the red‐green spectrum, with PVA + Ch + Np + Lp showing −1.11 ± 0.12, indicating a greenish tint. The b* parameter, which measures the yellow‐blue spectrum, showed positive values for yellows and negative for blues, characteristic of the PVA matrix (Firdaus et al. 2024).

**TABLE 4:** Color parameters of PVA‐based bioactive films modified with chitosan (Ch), itaconic acid (IA), nanoparticles (Np), and lemon peel extract (Lp).

	L*	a*	b*	C*	ΔE	Hue angle (°)	Color index
PVA	35.99 ± 0.63	0.14 ± 0.11	−1.00 ± 0.28	1.02 ± 0.27	0.00 ± 0.00	180 ± 1.47	−3.96 ± 0.44
PVA + Np + Lp	35.87 ± 0.28^ns^	−0.40 ± 0.61^ns^	0.98 ± 0.17*	1.16 ± 0.24^ns^	2.13 ± 0.25*	244.2 ± 0.98***	−11.28 ± 0.28***
PVA + IA	36.85 ± 0.25^ns^	0.08 ± 0.04^ns^	−0.85 ± 0.13^ns^	0.86 ± 0.13^ns^	0.88 ± 0.23^ns^	184.6 ± 2.55***	−2.66 ± 0.19^ns^
PVA + IA + Np + Lp	38.17 ± 0.11*	−0.10 ± 0.31^ns^	0.51 ± 0.24*	0.57 ± 0.23^ns^	2.68 ± 0.15**	225.5 ± 3.01***	−5.34 ± 0.36^ns^
PVA + Ch	36.32 ± 0.13^ns^	−0.59 ± 0.54^ns^	−0.30 ± 0.21^ns^	0.68 ± 0.53^ns^	1.16 ± 0.22^ns^	206.4 ± 2.65***	54.12 ± 0.99***
PVA + Ch + Np + Lp	35.17 ± 0.74^ns^	−1.11 ± 0.12^ns^	10.01 ± 0.80***	10.07 ± 0.79***	11.13 ± 0.75***	191 ± 1.48***	−3.15 ± 0.14^ns^

*Note:* The results are expressed as mean values ± SD (*n* = 3). A two‐way ANOVA followed by Tukey's multiple comparisons test was performed, with *p*‐values indicated as follows: ***p < 0.001, **p < 0.01, *p < 0.05, and ns (not significant) for *p* > 0.05.

The hue angle indicates dominant color families, with pure PVA films having a hue angle of 180° ± 1.47°. Additions of Lp and Ch shifted the hue to 191.0° ± 1.48° and 244.2° ± 0.98°, suggesting a greenish tint. IA‐containing films demonstrated moderate hue shifts due to interactions from IA's hydrophilic groups. SEM micrographs also revealed smoother, denser surfaces in IA‐ and Ch‐based films, which may potentially reduce diffuse reflection and alter color perception.

Chroma measures color saturation, with PVA + Ch + Np + Lp achieving the highest chroma (10.07 ± 0.79), while PVA + IA + Np + Lp had the lowest (0.57 ± 0.23), indicating reduced color intensity. The color index, with PVA IA at −2.66 ± 0.19 and pure PVA at −3.96 ± 0.44, shows a less chromatic appearance in both cases. Overall, incorporating Ch, Np's, and Lp extract significantly affected the optical properties of PVA‐based films. The observed changes in hue, chroma, and color index reflect complex interactions among components, enhancing functionality and aesthetic appeal crucial for active food packaging applications (Zeng et al. 2023).

### 3.4 Biodegradability Assessment

**FIGURE 7:**
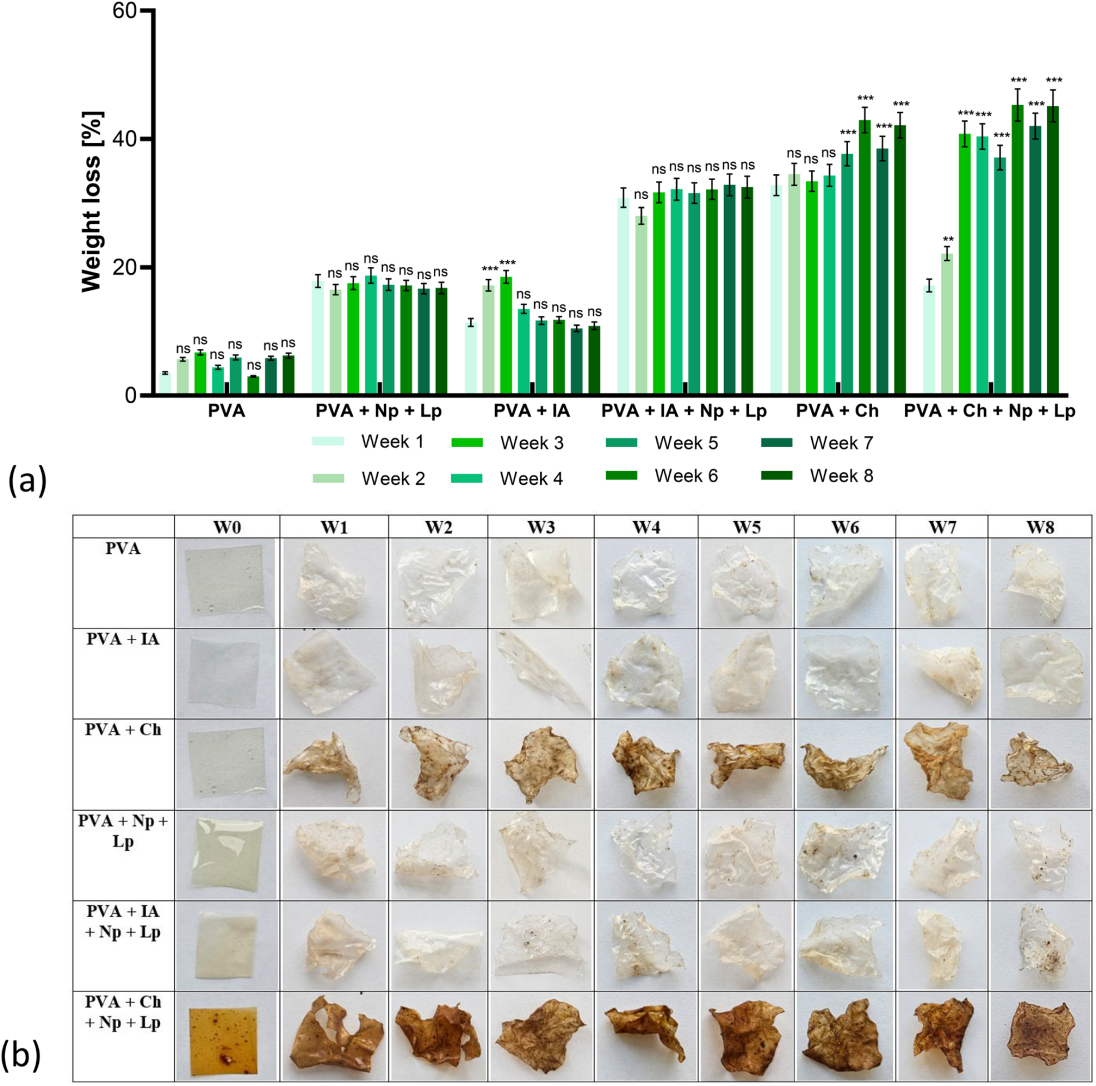
(a) Weight loss (over 8 weeks), (b) visual representation of the biodegradability images of bioactive films. The results are expressed as mean values ± SD (*n* = 3). A two‐way ANOVA followed by Tukey's multiple comparisons test was performed, with *p*‐values indicated as follows: ***p < 0.001, **p < 0.01 and ns (not significant) for *p* > 0.05.

Over an eight‐week period, the biodegradability of PVA‐based bioactive films was assessed through weight loss and visual disintegration in a soil burial system (Figure 7a). Weight loss data revealed significant differences among formulations, with values ranging from 25.4% ± 2.1% (PVA) to 61.8% ± 1.8% (PVA + Ch + Np + Lp) by Week 8. The pure PVA film exhibited the slowest degradation, with weight loss increasing steadily from 5.3% ± 0.4% in Week 1 to 25.4% ± 2.1% by Week 8. Its semi‐crystalline structure limited microbial attack, as observed in similar studies (Firdaus et al. 2024). Conversely, films containing IA degraded more rapidly, with weight loss reaching 39.6% ± 1.7% by Week 8. The enhanced biodegradability is attributed to IA's hydrophilic carboxylic groups, which facilitate microbial colonization, consistent with the findings by Xue et al. (2024).

The PVA + Ch film exhibited significant degradation (*p < 0.05), with weight loss of 50.4% ± 1.5% by Week 8. Ch's polysaccharide structure facilitated enzymatic hydrolysis by soil microorganisms, supporting previous results from do Nascimento et al. (2024) (do Nascimento et al. 2024). Visible fragmentation was observed by Week 4, progressing to substantial disintegration by Week 8. Incorporating Np's and Lp extract (PVA + Np + Lp) further improved biodegradability, with a weight loss of 34.8% ± 1.3% by Week 8. The bioactive compounds in Lp, such as pectin and polyphenols, enhanced microbial activity, as reported by Saleem et al. (2023). These films also showed moderate visual degradation and were partially fragmented by Week 5 (Saleem et al. 2023).

The PVA + IA + Np + Lp film demonstrated intermediate degradation, with weight loss reaching 45.7% ± 1.9% by Week 8. The combined effects of IA's hydrophilicity, Np's structural contribution, and Lp's bioactive properties enhanced microbial interactions. This is consistent with Shi et al. (2024), who noted faster degradation in pectin‐based films reinforced with bioactive components (Shi et al. 2024). The most rapid degradation occurred in PVA + Ch + Np + Lp films, with weight loss reaching 61.8% ± 1.8% by Week 8. The synergistic effects of Ch's enzymatic degradability, Lp's microbial stimulation, and Np's structural impact created a highly degradable matrix. Jayakumar et al. (2023) reported similar results, where Ch and bioactive extracts significantly enhanced soil biodegradability (Jayakumar et al. 2023).

Visual observations confirmed the weight loss trends shown in Figure 7b. The PVA film exhibited minimal discoloration and fragmentation, while films containing IA and Lp showed early disintegration by Week 4. The PVA + Ch + Np + Lp films degraded extensively, leaving only minor remnants by Week 8. These results demonstrate the potential of bioactive PVA‐based films as environmentally sustainable packaging materials. Adding Ch, IA, Np's, and Lp significantly accelerates biodegradation, making these films suitable for reducing environmental waste while maintaining initial functionality. While PVA is classified as a synthetic polymer, it is regarded as biodegradable under specific environmental conditions, particularly in the presence of activated sludge, soil microbes, or through oxidative degradation processes. Previous research has shown that PVA can achieve notable biodegradation rates of over 60% within 30 to 60 days when subjected to controlled compost or soil environments (Oun et al. 2022). In our study, films incorporating PVA exhibited up to 61.8% degradation within an 8‐week period, which supports its potential for biodegradability.

The study highlights the benefits of incorporating Lp extract, Np's, IA, and Ch into bioactive films. However, variations in processing conditions may affect their effectiveness. While the research focuses on specific properties of the biofilms, it does not fully address all variables or practical challenges related to real‐world applications and scalability. Although biodegradability has been tested, the research does not provide oxygen permeability, and neither does it include a complete life cycle assessment of the films, including energy inputs and waste generation during production. Additionally, the synthesized Np's may be safe for food‐related applications at appropriate concentrations, but their toxicity needs to be assessed in the future. Furthermore, a sensory evaluation (hedonic test) and a toxicity study would have been beneficial to better evaluate consumer acceptance and safety. These aspects are suggested as important directions for future exploration.

## 4 Conclusions

This study demonstrates that bioactive coatings can effectively preserve the quality and extend the shelf life of perishable foods, particularly blueberries, with potential applications for other fruits pending further testing. PVA‐based bioactive films significantly enhanced firmness and reduced microbial growth, maintaining blueberry visual and structural integrity for up to 11 days under refrigerated storage. These coatings improved the mechanical properties, while tensile strength increased to 10.7 MPa in PVA + Ch films and elevated antioxidant activity, achieving up to 90.36% inhibition in PVA + Ch + Np + Lp formulations. Additionally, they reduced water vapor permeability to 1.6% ± 1.2% and improved aesthetic properties through color stabilization.

The films displayed notable biodegradability, decomposing by up to 61.8% in soil within 8 weeks, emphasizing their environmental benefits. These findings suggest that bioactive coatings can preserve the quality of ready‐to‐eat blueberries while promoting more sustainable packaging practices. Future research will explore the life cycle assessment of the films, the safety of Np‐incorporated films for food contact compliance, and sensory properties, such as taste and appearance, to optimize scalability, cost efficiency, and consumer acceptability for commercial application.

## Author Contributions

**Bernadette‐Emőke Teleky:** conceptualization (equal), resources (lead), software (equal), writing – original draft (lead). **Silvia Amalia Nemes:** methodology (equal), writing – review and editing (equal). **Diana Plamada:** writing – review and editing (equal). **Laura Mitrea:** conceptualization (equal), writing – original draft (equal). **Calina Ciont:** conceptualization (supporting), writing – review and editing (equal). **Mădălina Medeleanu:** formal analysis (supporting), writing – original draft (equal). **Mădălina‐P. Plosca:** investigation (equal), writing – original draft (supporting). **Bianca Obreja:** investigation (supporting), writing – review and editing (supporting). **Răzvan Odocheanu:** formal analysis (equal), writing – review and editing (equal). **Loredana Leopold:** investigation (supporting), software (supporting). **Lucian Barbu‐Tudoran:** investigation (equal), software (equal). **Floricuța Ranga:** investigation (equal), software (equal). **Dan C. Vodnar:** funding acquisition (supporting), supervision (equal), writing – review and editing (equal).

## Ethics Statement

This study does not involve any human or animal testing.

## Conflicts of Interest

The authors declare no conflicts of interest.

## Data Availability

Data will be made available on request.
